# Combined effects of bone morphogenetic protein-7 and mineral trioxide aggregate on the proliferation, migration, and differentiation of human dental pulp stem cells

**DOI:** 10.1590/1678-7757-2022-0086

**Published:** 2022-09-09

**Authors:** Selen KÜÇÜKKAYA EREN, Elham BAHADOR ZIRH, Selim ZIRH, Parisa SHARAFI, Naciye Dilara ZEYBEK

**Affiliations:** 1 Hacettepe University Faculty of Dentistry Department of Endodontics Ankara Turkey Hacettepe University, Faculty of Dentistry, Department of Endodontics, Ankara, Turkey.; 2 TOBB University of Economics and Technology Faculty of Medicine Department of Histology and Embryology Ankara Turkey TOBB University of Economics and Technology, Faculty of Medicine, Department of Histology and Embryology, Ankara, Turkey.; 3 Erzincan Binali Yıldırım University Faculty of Medicine Department of Histology and Embryology Erzincan Turkey Erzincan Binali Yıldırım University, Faculty of Medicine, Department of Histology and Embryology, Erzincan, Turkey.; 4 TOBB University of Economics and Technology Faculty of Medicine Department of Medical Biology and Genetics Ankara Turkey TOBB University of Economics and Technology, Faculty of Medicine, Department of Medical Biology and Genetics, Ankara, Turkey.; 5 Hacettepe University Faculty of Medicine Department of Histology and Embryology Ankara Turkey Hacettepe University, Faculty of Medicine, Department of Histology and Embryology, Ankara, Turkey.

**Keywords:** Calcium silicate, Cytotoxicity, Osteogenic protein-1, Pulpotomy, Regenerative endodontics

## Abstract

Bioactive molecules present the potential to be used along with biomaterials in vital pulp therapy and regenerative endodontic treatment.

**Objective:**

The aim of this study was to assess the effects of the combined use of bone morphogenetic protein-7 (BMP-7) and mineral trioxide aggregate (MTA) on the proliferation, migration, and differentiation of human dental pulp stem cells (DPSCs).

**Methodology:**

For the proliferation analysis, DPSCs were incubated with a growth medium and treated with MTA and/or BMP-7 at different concentrations. For the following analyses, DPSCs were incubated with a differentiation medium and treated with MTA and/or BMP-7. Moreover, there were groups in which DPSCs were incubated with the growth medium (control), the differentiation medium, or DMEM/F12 containing fetal bovine serum, and not treated with MTA or BMP-7. Cell proliferation was analyzed using the WST-1 assay. The odontogenic/osteogenic differentiation was evaluated by immunocytochemistry, alkaline phosphatase (ALP) activity assay, alizarin red staining, and reverse transcription-quantitative polymerase chain reaction (RT-qPCR). Cell migration was evaluated using a wound-healing assay. Data were analyzed using analysis of variance and Tukey test (p=0.05).

**Results:**

The use of BMP-7 with MTA presented no significant effect on cell proliferation in comparison with the treatment with MTA alone (p>0.05), but showed higher ALP activity, increased mineralization, and higher expression of DMP1 and DSPP when compared with other groups (p<0.05). Nestin expression was higher in the control group than in groups treated with MTA and/or BMP-7 (p<0.05). The cell migration rate increased after treatment with MTA when compared with other groups in all periods of time (p<0.05). At 72 hours, the wound area was smaller in groups treated with MTA and/or BMP-7 than in the control group (p<0.05).

**Conclusion:**

The use of BMP-7 with MTA increased odontogenic/osteogenic differentiation without adversely affecting proliferation and migration of DPSCs. The use of BMP-7 with MTA may improve treatment outcomes by increasing repair and regeneration capacity of DPSCs.

## Introduction

The aim of vital pulp therapy (VPT), which includes indirect pulp capping, direct pulp capping, partial pulpotomy, and complete pulpotomy, is to preserve the vitality and health of the pulp tissue that may be affected by trauma, caries, or dental procedures.^1^ The success of VPT depends on the properties of the materials used in close proximity to the pulp.^2^ In recent years, the use of biomaterials in VPT has gained importance due to their high biocompatibility, bioactivity, and good sealing ability.^3^

Mineral trioxide aggregate (MTA), a calcium silicate-based biomaterial, is preferred by dentists to perform VPT due to its good sealing ability, biocompatibility, and bioactivity.^4^ MTA is associated with good clinical outcomes.^3^ However, the success of MTA varies depending on the pulp diagnosis and type of VPT performed.^5 - 7^ According to a previous systematic review, the success rate of direct pulp capping with MTA ranged from 61% to 100%,^5^ while the success rate of pulpotomy with MTA ranged from 83% to 100%.^8 - 10^ Despite the promising results, there is an ongoing search for materials and applications that achieve the best biological effects to improve the clinical outcomes of VPT.

Bioactive molecules present the potential to be used along with biomaterials in both VPT and regenerative endodontic treatment (RET).^11^ RET is generally performed by inducing apical bleeding to form a blood clot in the root canal and placing a biomaterial on the clot.^12^ These procedures release bioactive molecules from the dentin extracellular matrix that regulate intercellular signal transduction and play an important role in the repair and regeneration processes of the dentin–pulp complex.^13^ Bone morphogenetic protein-7 (BMP-7), also called osteogenic protein-1 (OP-1), is one of these bioactive molecules and can induce dental pulp stem cells (DPSCs) to undergo odontogenic differentiation.^14^ MTA can also induce odontogenic differentiation of DPSCs.^15^ This effect of MTA is not only related to its alkaline pH and the release of calcium ions, but also to its ability to induce the release of these bioactive molecules from dentin.^16^ The use of BMP-7 with MTA could improve the biological response and increase the clinical success of VPT and RET by enhancing the repair and regeneration processes. Therefore, the aim of this study was to assess the effects of the combined use of BMP-7 and MTA on the proliferation, migration, and differentiation of DPSCs. The null hypothesis was that the use of BMP-7 with MTA would have similar effects to the use of MTA on the proliferation, migration, and differentiation of DPSCs.

## Methodology

### Cell culture

Commercial human DPSCs (PT-5025, Lonza, Basel, Switzerland) were purchased. These cells were characterized and certified by the manufacturer.^17^ DPSCs were cultured in a growth medium (DPSC Bullet Kit PT-3005, Lonza, Basel, Switzerland), according to the manufacturer’s instructions.^17^ Cryopreserved cells were thawed, transferred to culture vessels (STARLAB, Hamburg, Germany), and incubated at 37°C with 5% CO_2_. The medium was changed every two days. Subculturing was performed when the cells reached about 80% confluency. Cells at passage 3 were used for all assays. $\text{Cells (}\left.3\times 10^{4}/\text{well}\right)$ were cultured in 24-well plates in the growth medium for 24 hours for each assay. Untreated DPSCs cultured in the growth medium served as a control for the experiments. All experiments were conducted in triplicate.

### Preparation of materials

MTA (ProRoot MTA, DENTSPLY Tulsa Dental Specialties, Johnson City, USA) was prepared by mixing 1 g MTA powder with 0.3 mL of distilled water, following the manufacturer’s instructions, and placed in sterile cylindrical polyethylene molds 2 mm high and 5 mm wide. Materials were stored at 37°C and 100% relative humidity for 24 hours. They were then removed from the molds and sterilized with ultraviolet light for one hour.

Recombinant human BMP-7 (4579-10, BioVision, San Francisco, USA) was prepared at various concentrations (25, 50, and 100 ng/mL) for the proliferation assay to establish the optimum concentration for the remaining assays.

### Proliferation assay

DPSC cultures were allocated into the following experimental groups and incubated for one, three, and five days:

Group MTA


Group MTA+BMP-7(25ng/mL)

Group MTA+BMP-7(50ng/mL)

Group MTA+BMP-7(100ng/mL)

Group BMP-7 (25ng/mL)

Group BMP-7 (50ng/mL)

Group BMP-7 (100ng/mL)

The prepared MTA samples were placed in transwells with 8 µm pore polycarbonate membrane inserts (Corning, Sigma-Aldrich, USA), which were immediately placed in culture plates containing the growth medium and DPSCs. The growth medium and recombinant human BMP-7 were refreshed every two days. After the incubation periods, each insert was removed, and the medium was withdrawn from each well and transferred to 96-well plates. An amount of 10 μL of WST-1 (Roche Applied Science, Penzberg, Germany) was added to each well and incubated for four hours at 37°C with 5% CO_2_. The optical density (OD) was measured at 450 nm on a microplate reader (VersaMax microplate reader, Molecular Devices, LLC, San Jose, USA). Cells cultured in the growth medium without MTA and BMP-7 served as a control group. The percentage of relative cell viability was calculated according to the following formula: (optical density of the experimental group/optical density of the control group)×100 .

### Odontogenic/osteogenic differentiation of DPSCs

**Figure 1:**
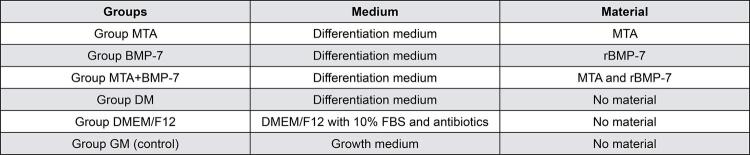
Group allocation according to the type of medium and material incubated with DPSCs DMEM/F12: Dulbecco’s modified eagle medium/nutrient mixture F12 DPSCs: Dental pulp stem cells, FBS: Fetal bovine serum MTA: Mineral trioxide aggregate rBMP-7: Recombinant human bone morphogenetic protein-7

$\text{DPSCs (}\left.3\times 10^{4}/\text{well}\right)$ were cultured in a differentiation medium (DM) containing Dulbecco’s modified eagle medium/nutrient mixture F12 (DMEM/F12) (Capricorn, Ebsdorfergrund, Germany) supplemented with 0.1 μM dexamethasone (D4902-100MG, Sigma-Aldrich, USA), 50 μM L-ascorbic acid (A4403-100MG, Sigma-Aldrich, USA), 10 mM glycerophosphate (G9422-50G, Sigma-Aldrich, USA), 10% fetal bovine serum (FBS-12B, Capricorn, Ebsdorfergrund, Germany), 100 U/mL penicillin, and 100 μg/mL streptomycin (PS-B, Capricorn, Ebsdorfergrund, Germany). DPSC cultures were allocated into groups, as Figure 1 shows, and incubated for 21 days.

The prepared MTA samples were placed in transwells with 8 µm pore polycarbonate membrane inserts (Corning, Sigma-Aldrich, USA), which were immediately placed in culture plates containing DPSCs. All culture mediums and recombinant human BMP-7 were refreshed every two days. The cell morphology of each group was assessed and recorded in the incubation period using an inverted microscope (DMI4000B, Leica, Wetzlar, Germany). To evaluate the odontogenic/osteogenic differentiation of DPSCs, immunocytochemical analysis, alizarin red staining, alkaline phosphatase (ALP) activity assay, and reverse transcription-quantitative polymerase chain reaction (RT-qPCR) analysis were performed at the end of the 21-day incubation period.

### Immunocytochemistry

The immunocytochemical analysis was performed by the markers dentin matrix protein 1 (anti-DMP1, bs-12359R, Bioss, USA), dentin sialophosphoprotein (anti-DSPP, bs-10316R, Bioss, USA), and nestin (anti-nestin, bs-0008R, Bioss, USA). Primary antibodies were diluted to 1:200 for all primer antibodies. DPSCs were seeded on 8-well chamber slides (10,000 cells/well). After the differentiation process, cells were fixed with methanol. For double-labeling, cells were incubated with the first primary antibody (anti-nestin) overnight at 4°C, followed by its specific secondary antibody (goat anti-mouse FITC, 1:1000, F9006, Sigma-Aldrich, USA). Thereafter, cells were incubated with one of the second primary antibodies (anti-DMP1 or anti-DSPP) and its specific secondary antibody (donkey anti-rabbit Alexa Fluor 568, 1:1000, A10042, Invitrogen, Carlsbad, CA, USA). Sections were mounted with fluorescence mounting medium containing DAPI (P36962, Thermo Fisher Scientific, Waltham, MA, USA). Stained cells were visualized using a light microscope with fluorescence attachment (DMI6B, Leica, Wetzlar, Germany).

### Quantification of immunofluorescence

All images were obtained by a microscope (DMI6B, Leica, Wetzlar, Germany) equipped with a camera (DFC7000T, Leica, Wetzlar, Germany). Images were analyzed using a software program (Image J bundled with 64-bit Java 1.8.0_172, NIH, USA), by selecting one cell at a time in an image and measuring the area, mean gray value, and integrated density.^18^ The fluorescence intensity was calculated according to the following formula: corrected total cell fluorescence (CTCF)=integrated density−(mean fluorescence of background readings×area of the selected cell). .

### Alizarin red staining

Alizarin red staining was performed to evaluate calcium deposition in cell culture. The medium was removed and cells were fixed with 4% formaldehyde for 15 minutes at RT. Then, cells were washed with distilled water and incubated with an Alizarin Red S solution (Sigma-Aldrich, USA) for 30 minutes at RT. Cells were washed five times with distilled water to remove the dye and assessed using an inverted microscope (DMI4000B, Leica, Wetzlar, Germany). Images were captured. To quantify the amount of mineral deposition, 10% acetic acid was added to each well and incubated for 30 minutes at RT. Cells were transferred to a microcentrifuge tube, vortexed for 30 seconds, and heated at 85°C for ten minutes. Tubes were incubated on ice for five minutes and centrifuged at 20,000 g for 15 minutes. The supernatant was transferred to a new tube and 10% ammonium hydroxide was added for neutralization. Samples were transferred to a 96-well plate. Absorbance was measured at 405 nm in the microplate reader (VersaMax microplate reader).

### ALP activity

ALP activity was analyzed by measuring the amount of p-nitrophenol (PNP) production using a colorimetric assay kit (BioVision, Milpitas, USA). To measure intracellular ALP activity, test samples and standards were prepared, following the manufacturer’s instructions. Washed cells were homogenized in assay buffer and centrifuged at 13,000 g for three minutes. Samples were distributed in a 96-well plate in equal volumes, by adjusting the total volume to 80 μL with assay buffer, and the p-nitrophenyl phosphate (PNPP) solution was added to each well. Standards were prepared in separate wells by diluting the PNPP solution with assay buffer, and 10 µL of ALP enzyme solution was added to these wells. The plate was incubated for 60 min at 25°C and protected from light. Finally, 20 µL of stop solution was added to each well to stop all reactions. Absorbance was measured at 405 nm in the microplate reader (VersaMax microplate reader). The PNP standard curve was plotted and the amount of PNP produced was calculated by applying sample readings to the standard curve. ALP activity was calculated according to the formula: ALP activity (U/mL)=amount of PNP (μmol)/volume of sample added in each well (mL)/reaction time (min) .

### RT-qPCR

The total RNA was isolated from cells using the RNeasy Mini kit (Qiagen, Maryland, USA) and the complementary DNA (cDNA) was obtained from purified RNA by ProtoScript^®^ II Reverse Transcriptase (New England Biolabs, Ipswich, USA). The synthesized cDNA was used as a template for quantitative real-time PCR in triplicate for each sample using the QuantiTect SYBR Green PCR Kit (Qiagen, Maryland, USA). The relative gene expression of DMP1, DSPP, and nestin was quantified, compared with the level of the housekeeping gene glyceraldehyde-3-phosphate dehydrogenase (GAPDH). The primer sequences used in this study were DMP1 (F: CGGAGGGTAGAGGTATCACAC, R: GCCTGTTCCTCTGAGCTAACTT), DSPP (F: GGGAATATTGAGGGCTGGAA, R: TCATTGTGACCTGCATCGCC), nestin (F: TCAAGATGTCCCTCAGCCTGGA, R: AAGCTGAGGGAAGTCTTGGAGC), and GAPDH (F: CATCACCATCTTCCAGGAG, R: AGGCTGTTGTCATACTTCTC). The reaction was performed in a Rotor Gene-Q thermocycler (Qiagen, Maryland, USA). The thermal cycling conditions were 95°C for five minutes followed by 40 cycles of 95°C for ten seconds, 60°C for 30 seconds, and 72°C for ten seconds. The relative gene expression was quantified after normalizing against the expression level of the housekeeping gene (GAPDH) as an internal control. The comparison of gene expression results was analyzed by calculating 2-ddCt values.

### Wound healing

The capability of cells to migrate was assessed by a wound-healing assay. $\text{DPSCs}\left(10\times 10^{4}/\text{well}\right)$ were seeded in 6-well culture plates and incubated for 24 hours to obtain a cell monolayer with approximately 90% confluence. A scratch was made manually with a sterile 200 μL pipette tip in each well and washed with phosphate-buffered saline to remove debris. DPSCs were incubated with test materials according to the groups. A phase-contrast microscope (DMI4000B, Leica, Wetzlar, Germany) was used to capture images at 0, 24, 48, and 72 hours to quantify and compare the cell migration rate. An image analysis software program (LASX, Leica, Wetzlar, Germany) was used to calculate the area with and without cells and the wound closure rate over time for each group was presented as a percentage.

### Statistical analysis

Analyses were performed using a software program (SPSS 22 for Windows, SPSS Inc., Chicago, USA). Data were presented as mean ± standard deviation. Two-way analysis of variance (ANOVA) and Tukey test were used to compare the cell proliferation assay data. Data of the remaining assays were analyzed using one-way ANOVA and Tukey test. The p-value<0.05 was considered statistically significant.

## Results

### Cell proliferation

**Figure 2:**
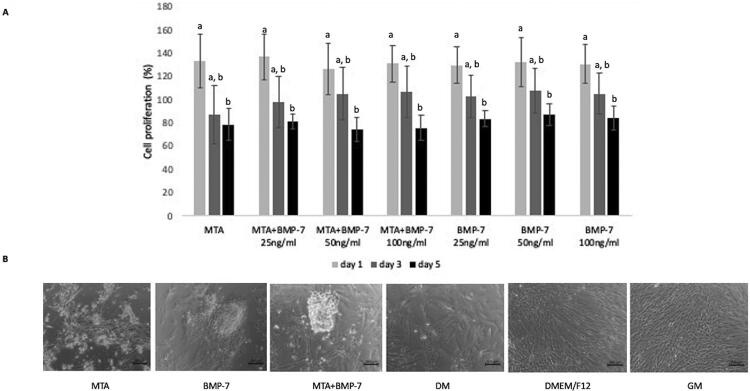
DPSC proliferation and differentiation. A) The percentage of cell proliferation after treatment with MTA and/or different concentrations of BMP-7. Data are presented as percentages of the control group. Different letters show statistically significant differences between groups (p<0.05). B) Inverted microscope images showing the morphology of cells in groups after 21 days of incubation. Cells in groups GM and DMEM/F12 presented a fibroblast-like morphology, while cells in other groups showed mainly nodule-like structures

Figure 2A shows the effect of adding different concentrations of recombinant BMP-7 on the proliferation of DPSCs treated with MTA for one, three, and five days. There were no significant differences among groups regardless of periods of time (p>0.05). The cell proliferation was significantly lower at five days than at one day in all groups (p<0.05). Therefore, the selected optimum dose of recombinant BMP-7 to be applied in further experiments was 50 ng/mL since the BMP-7 concentration did not cause a significant cytotoxic effect on DPSCs.

### Immunocytochemistry

**Figure 3:**
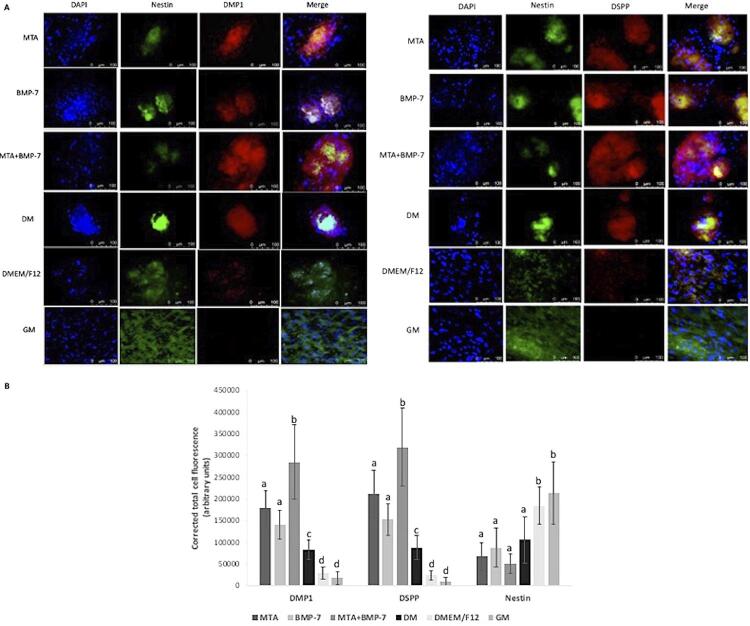
DMP1, DSPP, and nestin expression of cells in groups. A) Double labeling immunofluorescence showing cytoplasmic expression of nestin (green), DMP1 (red, left), and DSPP (red, right). Nuclei were counterstained with DAPI (blue). Scale bars=100 mm. B) Quantification of the immunofluorescence intensity in groups. Different letters show statistically significant differences between groups for each protein (p<0.05)

After 21 days of incubation, cells in groups DMEM/F12 and GM (control) presented a fibroblast-like morphology, while in other groups, cells formed nodule-like structures, showing differentiation ( Figure 2B ). DMP1 and DSPP increased in groups in which DPSCs were cultured in the differentiation medium, while nestin expression was predominant in groups DMEM/F12 and GM ( Figure 3A ).

The quantification of fluorescent images revealed that the highest expression of DMP1 and DSPP was in group MTA+BMP-7 (p<0.05), while the lowest expression was in groups GM and DMEM/F12 (p<0.05). Nestin expression in groups presented an opposite profile to DMP1 and DSPP. Quantification of immunofluorescence results are presented in Figure 3B .

### Alizarin red staining

**Figure 4:**
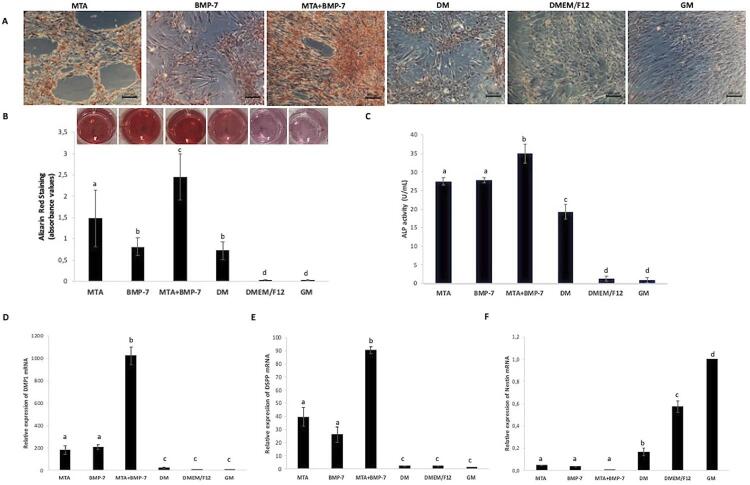
The odontogenic/osteogenic potential of cells in groups. A) Inverted microscope images of cells in groups after alizarin red staining. B) Image showing alizarin red staining of cultured cells in plates. Quantitative analysis of mineralized deposits in cultures. C) ALP activity analysis results. RT-qPCR results showing the relative gene expression of D) DMP1, E) DSPP, and F) nestin. Different letters show statistically significant differences between groups (p<0.05)

Images obtained after alizarin red staining showed that the combined use of MTA and BMP-7 increased mineralization when compared with other groups ( Figure 4A ). According to these images, the level of mineralization was low when cells were incubated only with the differentiation medium. No mineral deposits were observed in groups GM and DMEM/F12. These findings were confirmed by quantitative analysis ( Figure 4B ).

### ALP activity

Figure 4C shows ALP activity assay results. Group MTA+BMP-7 presented the highest ALP activity (p<0.05). There was no significant difference regarding ALP activity between groups MTA and BMP-7 (p>0.05), but they showed higher activity than group DM (p<0.05). Groups GM and DMEM/F12 showed the lowest ALP activity, but there was no difference between them (p<0.05).

### RT-qPCR

The RT-qPCR analysis showed that the expression of DMP1 and DSPP was higher in differentiated groups when compared with group GM (p<0.05) ( Figures 4D and E ). Group MTA+BMP-7 presented the highest expression of DMP1 and DSPP mRNA (p<0.05) ( Figures 4D and 4E*)* . Nestin expression was lower in differentiated groups when compared with group GM (p<0.05) ( Figure 4F ).

### Wound healing

**Figure 5:**
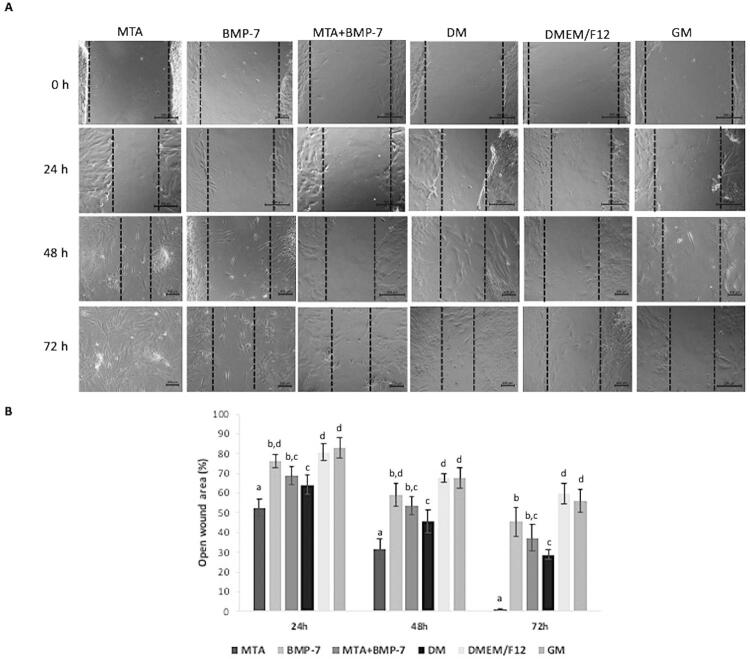
Wound healing assay results. A) Phase-contrast microscope images showing the healing over the scratch in groups over time. B) The wound area was calculated in percentage as the ratio of the residual wound area at the given period and the original wound area at zero hour. Different letters show statistically significant differences between groups for each period (p<0.05)

Figure 5 presents wound healing analysis results. The healing over the scratch increased significantly after treatment with MTA when compared with other groups at all periods of time (p<0.05). Cell migration rates in groups BMP-7 and MTA+BMP-7 were similar over time (p>0.05). At 72 hours, groups DMEM/F12 and GM presented the largest voids when compared with other groups (p<0.05).

## Discussion

The ideal material for both VPT and RET should promote repair and regeneration by stimulating cell proliferation, migration, and differentiation.^19^ Although MTA can induce dentinogenesis to some extent, its use along with various bioactive molecules may increase this potential.^20^ This study assessed the biological effects of the use of MTA and BMP-7 on DPSCs. The null hypothesis was partially accepted as the use of BMP-7 with MTA presented similar effects on cell proliferation to the use of MTA alone. However, MTA and BMP-7 decreased the cell migration rate while increasing the odontogenic/osteogenic differentiation of DPSCs when compared to MTA.

The selection of markers is essential for the assessment of the odontogenic/osteogenic differentiation, and a combination of markers, such as dentin sialoprotein (DSP), dentin phosphoprotein (DPP), DMP1, and nestin, has been recommended for this assessment.^21^ Therefore, DSPP, DMP1, and nestin were selected as odontogenic/osteogenic differentiation markers in this study. DMP1 is an acidic phosphoprotein mainly present in dentin, bone, and cementum.^21^ DSPP, a phosphorylated non-collagenous protein, which is cleaved into DSP and DPP, is highly expressed in odontoblasts.^21^ Nestin is an intermediate filament predominantly expressed in the developing nervous system.^22^ It has been reported that DPSCs that highly express nestin present a significant ability to show neurogenic differentiation.^23^ On the other hand, according to previous studies, nestin presents a potential role in odontoblast differentiation, as functional odontoblasts also express nestin.^22 , 24^ In this study, although the nestin expression was present, it was lower in groups cultured in the differentiation medium. DMP1 and DSPP expression, in turn, were higher in these groups, showing the odontogenic/osteogenic differentiation. This result can be interpreted as the mechanism of the odontogenic/osteogenic differentiation of human DPSCs associated with high DMP1 and DSPP expressions and a low nestin expression, in line with previous studies.^25 , 26^

In an animal study, recombinant BMP-7 with collagen as a pulp capping material after pulpotomy resulted in periapical lesion formation.^27^ This is probably related to the inadequate sealing ability of recombinant BMP-7 when used without a repair material like MTA. In another animal study, BMP-7 was applied with MTA as a pulp capping material and hard tissue was formed under materials.^28^ However, the formed hard tissue was more similar to bone than to dentin in that study.^28^ On the other hand, in our study, DPSCs cultured with MTA and BMP-7 presented higher DMP1 and DSPP expressions, showing the odontogenic/osteogenic differentiation. Furthermore, the use of BMP-7 with MTA increased ALP activity, which is an early indicator of the odontogenic/osteogenic differentiation of DPSCs, and enhanced alizarin red staining, which shows mineralized nodule formation, confirming the inductive effect of this combined use in mineralization and differentiation of DPSCs. The messenger RNA expression of DMP1 and DSPP also increased in cells treated with MTA and BMP-7.

On the other hand, the findings of this study regarding the wound healing assay showed that the incubation of cells with BMP-7 did not significantly affect cell migration when compared to the control group at 24 and 48 hours, similar to a previous study.^29^ Moreover, the use of BMP-7 with MTA resulted in slower cell migration in comparison with the use of MTA alone. This finding suggests that BMP-7 may greatly promote the odontogenic/osteogenic differentiation of DPSCs at the expense of cell migration.^29^ The treatment with MTA and BMP-7 may result in the selective recruitment of DPSCs by chemotactic cytokines to act as endogenous cell sources for mineralization.

Different results among studies may be related to different study designs, such as the type of contact between cell and material, the choice of the BMP-7 dose, and the method of BMP-7 application. Study designs regarding the relationship between cells and test materials, such as the direct contact method or the use of material extracts, may affect the cell response to the material.^30^ The use of material extracts may not allow a complete analysis of the cell–material interaction in the clinical environment, as MTA releases different amounts of molecules over time.^31^ According to a previous study, the direct contact of cells with MTA increased the differentiation into odontoblast-like cells.^32^ On the other hand, another study showed that its direct contact with MTA decreased cell viability and induced cell apoptosis.^33^ In laboratory conditions, the surface of culture plates usually serves as a good environment for cell attachment and proliferation, and any change in the surface may interfere in cell attachment and growth, affecting the study results.^34^ Therefore, MTA discs were placed in culture inserts to prevent direct physical interaction between MTA and cells, similar to previous studies.^34 , 35^ The insert system used in this study presented large pores that allowed soluble compounds from MTA to reach DPSCs over time.

The proliferation analysis was performed using WST-1, which is a tetrazolium salt that produces a highly water-soluble formazan by mitochondrial dehydrogenase enzymes.^36^ The WST-1 assay is a widely used colorimetric test to assess cellular viability and proliferation.^36^ The BMP-7 dose was selected according to the proliferation assay results. In differentiation assays, lower doses of BMP-7 may be insufficient to produce the aimed effects, while higher doses may trigger competing pathways that cause opposing effects on the downstream signaling cascades.^14^ As the BMP-7 dose did not significantly affect cell proliferation, 50 ng/mL was chosen as an optimum dose in this study. Regardless the BMP-7 dose, there was a decrease in cell proliferation at 72 hours of incubation with MTA, similar to previous studies.^37 , 38^ The decrease in cell proliferation may be the release of calcium hydroxide due to the hydration reaction of MTA.^33^ Although the release of calcium hydroxide causes an initial chemical irritation, it initiates the hard tissue production process with stem cell recruitment and differentiation.^33^ According to a previous study, a single application of recombinant BMP-7 can be insufficient to induce dentin formation.^39^ Proteins, including BMP-7, usually present low stability and degrade rapidly.^40^ For this reason, recombinant human BMP-7 was refreshed every two days in this study. This application method may have improved the odontogenic/osteogenic differentiation of DPSCs. In the clinical environment, this approach can be applied using delivery systems that allow the controlled and sustained release of BMP-7 doses.^40^

## Conclusion

The application of BMP-7 with MTA can be recommended during VPT and RET to enhance the repair and regeneration processes by improving the odontogenic/osteogenic differentiation of DPSCs.
